# Relationships between serum HER2 ECD, TIMP-1 and clinical outcomes in Taiwanese breast cancer

**DOI:** 10.1186/1477-7819-10-42

**Published:** 2012-02-17

**Authors:** Hsiu-Pei Tsai, Shin-Cheh Chen, Huei-Tzu Chien, Yi-Yin Jan, Tzu-Chieh Chao, Miin-Fu Chen, Ling-Ling Hsieh

**Affiliations:** 1 Graduate Institute of Clinical Medical Sciences, Chang Gung University, Tao-Yuan, Taiwan; 2 Department of General Surgery, Chang Gung Memorial Hospital, Tao-Yuan, Taiwan; 3 Graduate Institute of Biomedical Sciences, Chang Gung University, Tao-Yuan, Taiwan; 4 Department of Public Health, Chang Gung University, Tao-Yuan, Taiwan

**Keywords:** breast cancer, HER2 ECD, TIMP-1, enzyme-linked immunosorbent assay, prognosis

## Abstract

**Background:**

Serum levels of the extracellular domain of HER2/neu (HER2 ECD) have been demonstrated to be associated with clinical outcomes. A disintegrin and metalloproteinase-10, a sheddase of HER2/neu, can drive cancer progression and its activity is inhibited by tissue inhibitor of metalloproteinase-1 (TIMP-1). However, elevated TIMP-1 expression has been associated with a poor prognosis of breast cancer. Therefore, this study was performed to explore the relationships between serum HER2 ECD, TIMP-1 and clinical outcomes.

**Methods:**

One hundred and eighty-five female breast cancer patients, who received curative mastectomy without neo-adjuvant chemotherapy at Chang-Gung Memorial Hospital, were recruited with informed consent for this study. Pre-operative serum levels of HER2 ECD and TIMP-1 were measured using an enzyme-linked immunosorbent assay.

**Results:**

Twenty-three cases (12.4%) were classified HER2 ECD positive. HER2 ECD positivity was significantly associated with age, lymph node involvement, histological grade, estrogen receptor status, progesterone receptor status, tissue HER2/neu overexpression, and disease-free survival (DFS). In an age, stage, ER and HER2/neu status matched subgroup (N = 41), the serum level of TIMP-1 was significantly associated with HER2 ECD positivity and DFS.

**Conclusions:**

A high serum TIMP-1 was significantly associated with HER2 ECD positivity and a poorer DFS among Taiwanese primary breast cancer patients with HER2 overexpression.

## Background

Amplification or overexpression of HER2/neu, a 185 kDa transmembrane tyrosine kinase receptor, has been reported in 20-30% of invasive breast cancers (IBCs) [1]. It predicts a more aggressive clinical course such as a transition from *in situ *growth to invasion [2], aggressive disease progression and poor treatment response [3-5]. In addition, it has been shown that there is a high concordant HER2/neu status in paired primary tumor and distant metastatic lesions on analysis by both immunohistochemistry (IHC) and by fluorescence *in situ *hybridization (FISH) [6-9]. Therefore, HER2/neu status is an important diagnostic and prognostic biomarker and is also one of the most dependable criteria for the use of trastuzumab-based chemotherapy to treat breast cancer.

In addition to HER2/neu status of the tumor tissue, the extracellular domain (ECD) of HER2 (HER2 ECD), which is shed from the HER2/neu receptor after a proteolysis process, has been shown to show a better correlation with tumor burden, treatment response, disease-free status and overall survival than the full-length HER2/neu [10]. However, certain clinical studies have not supported baseline serum HER2 ECD as a reliable predictor of tumor progression, treatment response, duration of response, or time to progression in advanced/metastatic breast cancer [11,12]. Thus, in agreement with the 2007 and 2009 American Society of Clinical Oncology guidelines on the use of biomarkers in breast cancer [11,13], there is currently insufficient evidence to support the use of serum HER-2 ECD in the routine management of individual patients with breast cancer.

It has been demonstrated that extracellular matrix remodeling proteinases, such as matrix metalloproteinases (MMPs), play a key role in the invasion and metastasis of cancer cells [14-16]. Recent studies have indicated that members of a zinc-dependent family of proteinases related to the MMPs, namely disintegrin and metalloproteinases (ADAMs), are also involved in cancer progression [17]. A major level of control MMP functions occurs via their interaction with specific tissue inhibitors of metalloproteinases (TIMPs) [18]. Similar to MMPs, ADAM family members are also inhibited by specific TIMPs [17]. Among known MMPs and ADAMs, ADAM-10 has been shown to play an important role in the shedding of dozens of substrates that drive cancer progression, including HER2/neu [19,20]. Furthermore, HER2 ECD shedding can be inhibited by broad-spectrum metalloprotease inhibitors such as TAPI, batimastat, and TIMP-1 [19,21]. However, elevated TIMP-1 expression in human cancers, including breast cancer, has been associated with a decreased time to recurrence and a lower overall survival [22-26].

It is well known that there are significant ethnic disparities between western and eastern countries in terms of breast cancer with respect to cancer incidence, the frequency of BRCA1 and BRCA2 mutation, tumor biology and molecular subtypes [27-29]. In this context, information on the role of serum HER2 ECD in Asian breast cancer women is limited. Therefore, this study was performed to explore the relationships between serum HER2 ECD level, serum TIMP-1 level and clinical outcomes. Since MMP-2 and MMP-9 (gelatinase A and B) have been shown to be associated with breast cancer, expression of HER2/neu, and an unfavorable prognosis [15,16] and because TIMP-2 also seems to have cell growth promoting and anti-apoptotic activity [30], serum MMP-2, MMP-9 and TIMP-2 were also included in the present analysis.

## Methods

### Patients and Clinicopathological Information Collection

This study was approved by the Institutional Review Board, Chang Gung Memorial Hospital (CGMH). One hundred and eighty-five female breast cancer patients received curative mastectomy without neo-adjuvant chemotherapy during 2001 and 2002 in CGMH and were retrospectively recruited with informed consent from our clinical sample library for this study. In order to have reasonable case numbers for studying the relationships between serum HER2 ECD and TIMP-1, the present cohort was intentionally comprised of most cases with HER2 overexpression. In addition, 23 women who came to CGMH for health examination during the same period and who were without cancer or other critical chronic disease, were also recruited with informed consent as normal controls. Clinicopathological information on the 185 breast cancer patients, including age, menopause status, echo-determined tumor size, lymph node metastasis, stage, DNA ploidy, SBR tumor grade, estrogen receptor status (ER), progesterone receptor status (PR), and HER2/neu evaluated by immunohistochemistry, regimens of treatment, disease-free and overall survival time, were collected.

### Analysis of Serum HER2 ECD, MMP-2, MMP-9, TIMP-1 and TIMP-2

Serum was collected from the control individuals during their physical examination and from the breast cancer patients before surgery; the samples were then stored at -70°C until they were assayed. The level of serum HER2 ECD was determined by enzyme-linked immunosorbent assay (ELISA) at Department of Clinical Pathology, CGMH, using two monoclonal antibodies that recognized a distinct epitope of the ECD of the HER2 protein as described previously [31]. All samples were assayed in duplicate. Since the mean value of the serum HER2 ECD of the 23 normal controls was 8.9 ng/ml, we defined individuals with a HER2 ECD level higher than 8.9 ng/ml as HER2 ECD-positive.

Serum levels of MMP-2, MMP-9, TIMP-1 and TIMP-2 were measured using a Biotrak ELISA System (Amersham Pharmacia Biotech Inc., USA). All samples were assayed in duplicate. According to the manufacture's protocol, 100 μl of diluted serum samples were added to the assay and the levels of MMP-2, MMP-9, TIMP-1 and TIMP-2 were determined using appropriate peroxidase-conjugated anti- MMP-2, MMP-9, TIMP-1 and TIMP-2 antibodies. The reactions were stopped by the addition of 100 μl of 1 M sulfuric acid and absorbance of the product was read at 450 nm within 30 minutes.

### Statistical Analysis

Statistical analysis was performed using SPSS version 11.0 (SPSS, Chicago, IL). The associations between the clinicopathological parameters and the HER2 ECD status were examined using the χ^2^-squared test or Fisher's exact test. Wilcoxon Rank Sums test and Fisher's exact test were used to compare all clinicopathological categories between the HER2 ECD positive and negative patients. Serum protease levels in the matched groups were also analyzed by the same methods. Survival curves were constructed using the Kaplan-Meier method and compared with the log-rank test (univariate analysis). Multivariate analysis was carried out using the Cox risk proportion model. A two-sided value of *p *< 0.05 was considered statistically significant.

## Results

### Relationship between Baseline Serum HER2 ECD Levels and Clinicopathological variables

Pre-operative serum and complete clinicopathological information were retrospectively collected from 185 patients receiving curative mastectomy between 2001 and 2002. Using the cut-off serum HER2 ECD level of 8.9 ng/ml, which is the mean value for the 23 normal-control females, 23 (12.4%) patients had serum HER2 ECD levels designated as positive. As shown in Table 1 HER2 ECD positivity was significantly associated with being older (*p *= 0.002), having lymph node involvement (*p *= 0.010), having an advanced stage (*p *< 0.0001), having a high histological grade (*p *= 0.029), the presence of non-diploid tumor DNA (*p *= 0.033), having a negative ER (*p *= 0.0002), having a negative PR (*p *= 0.015) and HER2/neu overexpression (*p *= 0.003).

**Table 1:** The associations between serum ECD level and clinicopathological characteristics in 185 breast cancer patients

Clinical parameters	ECD negative N = 162	ECD positive N = 23	*ρ *value
**Age (year-old)**			
< = 50	91 (94.8%)	5 (5.2%)	0.002*
> 50	71 (79.8%)	18 (20.2%)	
**Tumor size (cm)**			
< = 3	114 (90.5%)	12 (9.5%)	0.080
> 3	48 (81.4%)	11 (18.6%)	
**Lymph node status**			
Negative	102 (92.7%)	8 (7.3%)	0.010*
Positive	60 (80.0%)	15 (20.0%)	
**Stage**			
0	13 (92.9%)	1 (7.1%)	< 0.0001*
I	37 (97.4%)	1 (2.6%)	
II	95 (90.5%)	10 (9.5%)	
III	17 (60.7%)	11 (39.3%)	
**SBR grade**			
Low	81 (92.0%)	7 (8.0%)	0.029*
High	52 (80.0%)	13 (20.0%)	
**DNA ploidy**			
Diploid	40 (95.2%)	2 (4.8%)	0.033*^a^
Non-diploid	69 (80.2%)	17 (19.8%)	
**Estrogen receptor status (ER)**			
Negative	93 (80.9%)	22 (19.1%)	0.0002*^a^
Positive^†^	69 (98.6%)	1 (1.4%)	
**Progesterone receptor status (PR)**			
Negative	107 (83.6%)	21 (16.4%)	0.015*^a^
Positive	55 (96.5%)	2 (3.5%)	
**HER2/neu expression (IHC)**			
Negative or Weak	43 (100.0%)	0 (0.0%)	0.003*^a^
Strong (> = 3+)	119 (83.8%)	23 (16.2%)	

*statistically significant at *p *< 0.05.

^†^Monoclonal antibody 6F11 was used as the primary antibody for ER IHC staining and the cutoff was set at 10% of tumor cells displayed staining to define "ER positive".

^a^by Fisher's exact test

**Figure 1:**
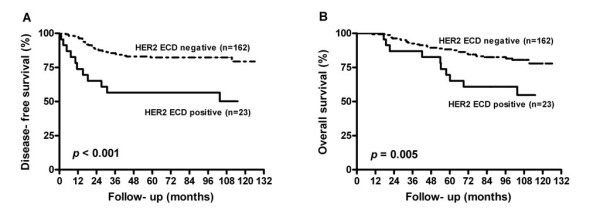
**Kaplan-Meier curves for disease-free survival and overall survival based on the analysis of serum HER2 ECD positivity**.

Disease-free survival and overall survival (DFS and OS) were significantly worse in patients that were serum HER2 ECD positive at diagnosis when compared with patients that were HER2 ECD negative (Figure 1). To assess the effect of the other clinicopathological variables and treatment on DFS and OS, a Cox model was carried out initially using univariate analysis. Being older (*p *= 0.037), lymph node involvement (*p *= 0.003), negative ER status (*p *= 0.0008) and non-hormone therapy (*p *= 0.001) were significantly associated with a poorer DFS (Table 2). Lymph node involvement (*p *= 0.002), negative ER status (*p *= 0.002) and non-hormone therapy (*p *= 0.001) were significantly associated with a poorer OS (Table 2). A multivariate Cox regression model for DFS and OS was then built using the variables that were found to be significant during the univariate analysis and lymph node involvement and negative ER status were significantly associated with poorer DFS and OS. In addition, hormone therapy would seem to improve DFS and OS (Table 2).

**Table 2:** Univariate and multivariate analysis of disease free survival and overall survival

Variables	Disease-free survival			Overall survival		
	
	*p *value			*p *value		
	Univariate	Multivariate	HR^a ^(95% CI)	Univariate	Multivariate	HR^a ^(95% CI)
Age	0.037	0.205^b^	1.53 (0.79 - 2.96)^b^	0.138		
		0.278^c^	1.44 (0.75 - 2.78)^c^			
Tumor size	0.862			0.563		
Lymph node	0.003	0.003^b^	2.70 (1.40 - 5.23)^b^	0.002	0.002^b^	2.81 (1.46 - 5.42)^b^
		0.008^c^	2.41 (1.25 - 4.64)^c^		0.005^c^	2.57 (1.34 - 4.93)^c^
ER	0.0008	0.004^b^	3.79 (1.53 - 9.38)^b^	0.002	0.004^b^	3.52 (1.50 - 8.23)^b^
PR	0.109			0.168		
HER2/neu	0.997			0.619		
Grade	0.183			0.318		
ECD	0.0002	0.214^b^	1.62 (0.76 - 3.47)^b^	0.005	0.451^b^	1.34 (0.62 - 2.90)^b^
		0.097^c^	1.89 (0.89 - 3.99)^c^		0.341^c^	1.45 (0.68 - 3.09)^c^
Chemotherapy*	0.122			0.224		
Radiation therapy	0.418			0.859		
Hormone therapy	0.001	0.011^c^	0.31 (0.13 - 0.77)^c^	0.001	0.004^c^	0.27 (0.11 - 0.65)^c^

* Anthracycline-based regimen (82/185, 44.3%), CMF (cyclophosphamide, methotrexate and 5-fluorouracil) (64/185, 34.6%), taxane (45/185, 24.3%), trastuzumab (22/185, 11.9%), lapatinib (3/185, 1.6%) and others (28/185, 15.1%) were used as the first line adjuvant chemotherapy in this breast cancer cohort.

^a^Adjusted for other prognostic variables as listed on the left.

^b, c^Since most patients who were ER positive underwent hormone therapy, ER and hormone therapy were included in the model separately.

### Relationship between Serum levels of MMPs, TIMPs and HER2 ECD

To determine the type of protease associated with higher serum HER2 ECD levels, the serum levels of two metalloproteases (MMP-2 and MMP-9) and two protease inhibitors (TIMP-1 and TIMP-2) were analyzed within a matched design. Age, stage, ER and HER2/neu status were used for matching to rule out their effects on HER2 ECD positivity (Table 1). In the end nineteen HER2 ECD positive samples and twenty-two HER2 ECD negative samples were enough for this analysis. As shown in Table 3 the serum level of TIMP-1 was significantly higher in HER2 ECD positive patients compared to HER2 ECD negative patients. However, there were no significant differences between HER2 ECD positive and negative patients in terms of their serum MMP-2, MMP-9, and TIMP-2 levels.

**Table 3:** Differences in serum MMP-2, MMP-9, TIMP-1 and TIMP-2 levels (ng/mL) in the ECD positive/negative patients

	ECD negative N = 22	ECD positive N = 19	**ρ value**^a^
MMP-2	685.09 ± 148.30	753.14 ± 219.17	0.359
MMP-9	39.85 ±29.97	40.82 ± 33.66	0.979
TIMP-1	242.45 ± 102.82	364.88 ± 139.38	0.006
TIMP-2	49.23 ± 21.81	62.92 ± 42.11	0.528

^a^by Wilcoxon two-sample test.

### Combined Effect of Serum TIMP-1 and HER2 ECD Levels on Clinical Outcomes

**Figure 2:**
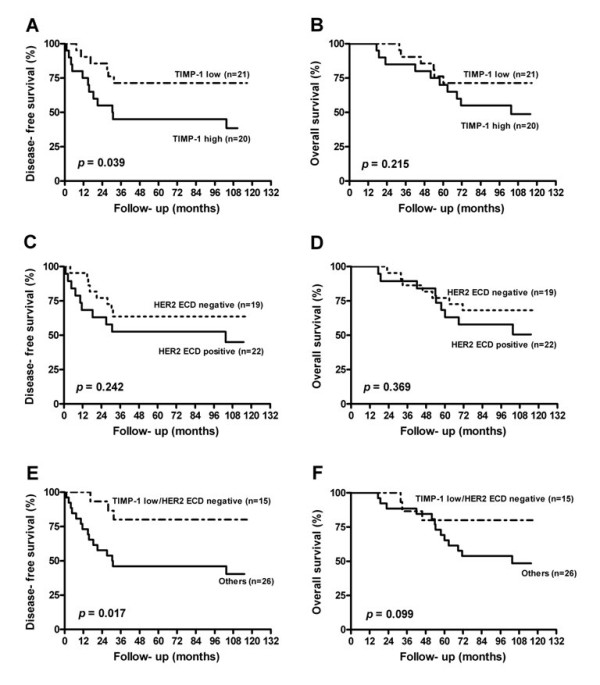
**Kaplan-Meier curves for disease-free survival and overall survival based on the analysis of serum TIMP-1 level (A, B), HER2 ECD positivity (C, D) and their combination (E, F) in 41 age, stage, ER and HER2/neu status matched patients**.

Since it has been reported that serum TIMP-1 was an independent predictive and prognostic factor for metastatic breast cancer [32], the effect of serum TIMP-1 and the combined effect of serum TIMP-1 and HER2 ECD on clinical outcomes was also explored using age, stage, ER and HER2/neu status matched patients (a total of 41 patients). The breast cancer patients were dichotomized into high and low TIMP-1 groups based on a median value of 270 ng/mL. Patients with a low TIMP-1 level and who were ECD negative were defined as the low-risk group, while the others were defined as the high-risk group. As shown in Figure 2, low serum TIMP-1 patients had significantly better DFS than high serum TIMP-1 patients (*p *= 0.039); however the effect of serum TIMP-1 on OS was insignificant (*p *= 0.215). Furthermore, the effects of serum TIMP-1 on clinical outcomes were strengthened when the serum HER2 ECD was taken into account.

## Discussion

The clinical roles of serum HER2 ECD in breast cancer have been widely discussed since this protein was first discovered. The percentage of serum HER2 ECD positivity has been found to be extremely variable in primary breast cancer patients. In our study, HER2 ECD positivity was detected in 12.4% (23/185). This rate is close to the mean value of the worldwide studies published between 1992 and 2007 [12], although the cut-off value in our study (8.9 ng/mL) is different from the value (15 ng/mL) cleared by the US Food and Drug Administration for use in the management of women with metastatic breast cancer [33].

High serum concentrations of HER2 ECD have been associated with HER2 overexpression [34], increased tumor burden [35], poorer survival [36] and resistance to endocrine therapy and chemotherapy [12,37,38], We found that HER2 ECD positivity was significantly associated with most clinicopathological parameters including tumor size, lymph node involvement, tumor stage, histological grade, chromosome ploidy, ER, PR, and HER2/neu membrane protein overexpression. These results imply that serum HER2 ECD levels may reflect the aggressiveness of the disease better than tissue HER2/neu receptor only. It is interesting to note that older patients had higher HER2 ECD positivity than younger patients in this series of patients. This phenomenon can be explained by the differences in the frequency of ER and PR negative tumors in different age groups of patients. In the present study, the frequencies of ER and PR negativity were significantly higher in older patients (70.8% and 83.2% for ER and PR, respectively) than younger patients (54.2% and 56.3% for ER and PR, respectively). The data from Taiwan Cancer Database also consistently shows that younger patients (≤50 years) have a higher prevalence of ER and PR expression compared with older patients (> 50 years) [27].

A high serum HER2 ECD level has been shown to act as an independent prognostic marker in one study by Ludovini et al. [39]. In the present study, serum HER2 ECD positivity was also associated with shorter DFS and OS in the univariate analysis. Probably due to a small number of positive HER2 ECD cases (n = 23), these associations were not significant in the multivariate analysis (Table 2). However, serum HER2 ECD positivity was a better predictor than HER2/neu status by IHC as indicated by previous studies [10,39].

The mechanism of HER2 ECD shedding from HER2/neu receptor is not been completely understood. It had been demonstrated that HER2 is one of the shedding substrates of ADAM-10 [19] and HER2 ECD shedding can be enhanced by fatty acid synthase [40] and inhibited by the broad-spectrum metalloprotease inhibitors such as TAPI, batimastat, and TIMP-1 [21]. In this study, we found that serum levels of TIMP-1 were significantly higher in HER2 ECD positive patients compared to HER2 ECD negative patients after correction for various clinicopathological factors (age, stage, ER and HER2/neu status). This observation was unexpected and contrary to the findings of the *in vitro *model system [21,41]. TIMP-1 is the prototypic inhibitor for most MMP family members [42]. Recent studies, however, have indicated that TIMP-1 also possesses a broad range of MMP independent biological activities including the induction of proliferation and the inhibition of apoptosis [43]. The induction of proliferation and the inhibition of apoptosis by TIMP-1 have been demonstrated to occur through activation of the phosphatidyl inositol-3 kinase (PI-3K) signaling pathway [44-46]. Furthermore, activation of PI-3K can lead to activation of phosphatidyl inositol-dependent kinase-1 (PDK1), which directly phosphorylates ADAMs and causes EGFR activation [47]. Therefore, it is possible that in some circumstances a high level of TIMP-1 may activate ADAM-10, which would directly lead to HER2 ECD shedding. The double-edged sword of TIMP-1 in terms of activation and inhibition of ADAM-10 activity and the effect of this on HER2 ECD shedding needs further investigation.

Since Talvensaari-Mattila et al. [26] reported that high serum TIMP-1 levels are significantly correlated with poorer relapse-free survival among breast cancer patients, several studies have obtained similar findings [48]. In our small matched study subjects (N = 41), adjuvant treatment was not different for the different TIMP-1 and HER2 ECD groups. We observed that high serum levels of TIMP-1 were associated with a poorer DFS, but not HER2 ECD (Figure 2). Furthermore, patients with a low TIMP-1 and a negative HER2 ECD had better clinical outcomes than the other combinations (Figure 2). This observation is similar to a study by Lipton et al. [49] They found that a combined analysis of both serum TIMP-1 and HER2/neu conferred an additional ability to predict subgroups of patients with significantly different clinical outcomes as compared to using either biomarker alone.

## Conclusions

A high serum TIMP-1 was significantly associated with HER2 ECD positivity and a poorer DFS among Taiwanese primary breast cancer patients with HER2 overexpression. Combined analysis of both serum TIMP-1 and HER2 ECD may have additional value when used for the clinical management of breast cancer patients with HER2 overexpression in Taiwan.

## Abbreviations

(HER2 ECD): extracellular domain of HER2/neu; (TIMP-1): tissue inhibitor of metalloproteinase-1; (DFS): disease-free survival; (IBCs): invasive breast cancers; (IHC): immunohistochemistry; (FISH): fluorescence *in situ *hybridization; (MMPs): matrix metalloproteinases; (ADAMs): a disintegrin and metalloproteinases; (ER): estrogen receptor; (PR): progesterone receptor; (ELISA): enzyme-linked immunosorbent assay; (OS): overall survival.

## Competing interests

The authors declare that they have no competing interests.

## Authors' contributions

SCC and LLH conceived the study. HPT performed ELISA assay. SCC, YYJ, TCC and MFC collected the cases and clinical information. HPT, SCC and LLH interpreted the ELISA. HPT, HTC and LLH performed the statistical analysis. HPT performed the literature review and wrote the manuscript. LLH supervised the experiments and manuscript writing. All authors read and approved the final manuscript.
